# Effect of Propolis on moderate persistent asthma: A phase two randomized, double blind, controlled clinical trial

**Published:** 2021

**Authors:** Majid Mirsadraee, Bahareh Azmoon, Shadi Ghaffari, Aboutaleb Abdolsamadi, Mohammad Reza Khazdair

**Affiliations:** 1 *Department of Internal Medicine, Faculty of Medicine, Islamic Azad University, Mashhad Branch, Mashhad, Iran*; 2 *Innovative Medical Research Center, Faculty of Medicine, Islamic Azad University, Mashhad Branch, Mashhad, Iran*; 3 *Shahid Hasehemi Nezhad Research Center, Kavosh High School, Ministry of Education, Mashhad, Iran*; 4 *Independent Pharmacist.*; 5 *Cardiovascular Diseases Research Center, Birjand University of Medical Sciences, Birjand, Iran*

**Keywords:** Propolis, Asthma, Caffeic acid phenethyl ester, Quercetin, Naringenin

## Abstract

**Objective:**

The aims of this study was to determine the effect of Propolis (resinous mixture that honey bees produce by mixing saliva and beeswax) on clinical and physiological findings of moderate persistent asthma.

**Materials and Methods:**

Fifty-two subjects aged 44.6±18.5 years old with moderate asthma and Forced expiratory volume in 1 second (FEV1) 60-79% of predicted, were enrolled in this clinical trial. We randomly allocated subjects to receive either propolis (75 mg three times a day) or a matched placebo for one month. Primary outcome was Asthma control test (ACT) score and secondary outcomes included dyspnea, spirometry, fractional exhaled nitric oxide (FENO) and sputum cytology including inflammatory cell. Sputum induction was done by hypertonic saline and cytology slides were stained by Papanicolaou stain.

**Results:**

Clinical findings significantly improved after the treatment. ACT scores significantly increased by using propolis (12.8±5.5 before and 18.1±4.99 after the trial), which was significantly higher than the placebo group (14.4±6.6 after the trial). The most significant physiological improvements were significant increases in FEV1, FV1/Forced vital capacity and expiratory flows. FENO showed significant decreases in the propolis group but increases in the placebo group. Cytological examination of sputum showed that the pattern of inflammation was eosinophilic in 44% subjects with an average eosinophil of 7.2±1.01%. Eosinophilia significantly decreased (p<0.05) by using propolis (7.2±1.01 and 4.3±3.1%, before and after treatment, respectively), but it significantly increased (p<0.04) in the placebo group (5.5±2.8, and 11.1±6.6%, before and after treatment, respectively).

**Conclusion:**

Propolis improved the clinical and physiological findings of moderate persistent asthma, and it was able to suppress eosinophilic inflammation.

## Introduction

The honey bee produces materials beside honey; one of them is propolis is collected from plants and used to reconstruct their hives (Sforcin, 2007). It is produced in very small amounts and is a source of bioflavonoid and triterpenes (precursors of steroids). Propolis is used in folk medicine for asthma. Recently, modern medicine has discovered its anti-inflammatory and antimicrobial effects (Sforcin, 2007). Some of the anti-inflammatory constituents of propolis are caffeic acid phenethyl ester (CAPE), caffeic acid (CA), quercetin and naringenin, as well as the synthetic compounds indomethacin (IM) and nordihydroguaiaretic acid (NDGA), and a novel lipoxygenase inhibitor N,N′-dicyclohexyl-O-(3,4-dihydroxycinnamoyl) isourea (DCHCU) (Mirzoeva and Calder, 1996). 

CAPE inhibited nucleous factor- Kappa B (NF-kB) and Nuclear factor of activated T cells (NFAT) production, and as a consequence decreased Interleukin-2 receptor (IL-2R) and T lymphocytic activity (Márquez et al., 2004; Wang et al., 2009). It also inhibited eotaxin and controlled eosinophils influx (Liao et al., 2010). Quercetin is another potent anti-inflammatory flavonoid that ameliorates asthma by reducing the eosinophilic mediators and type 2 helper cytokines and increases interferon gamma (IFNγ) (Park et al., 2009). Another study showed effective suppression of eosinophilic activity by 5 µM of quercetin (Sakai-Kashiwabara and Asano 2013). Naringenin showed effective anti-inflammatory ability in the murine model of asthma by lowering eosinophilic inflammation, CD4 T cell cytokine production and mucus production (Iwamura et al., 2010). It was also able to suppress IL-4, IL-13, NF-κB DNA-binding activity and level of chemokines including CCL5, CCL11, and inducible nitric oxide synthase (iNOS) (Shi et al., 2009). 

Although, in a murine model of asthma, propolis showed effective suppression of acute immunological and allergic reaction (Farias et al., 2014; Mirzoeva and Calder 1996; Sy et al., 2006) besides chronic phenomena causing remodeling of asthma (Kao et al., 2013), its efficacy to treat asthma was not widely evaluated in human beings. Recommended daily dosages of propolis is 1.4 mg/kg per day and dosages higher than 300 mg/day may cause inflammatory side effects and it is considered toxic at higher dosages (Sforcin 2007). Here a question arises: Does propolis suppress inflammation in asthma?

The aim of this study was to determine the efficacy of propolis in human asthma in a controlled clinical trial.

## Materials and Methods

**Participants**

**Figure 1:**
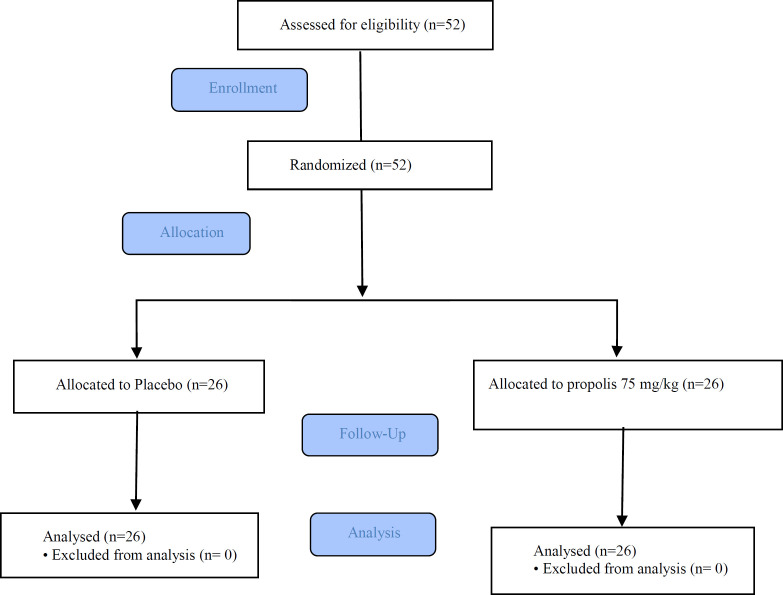
Flowchart of the study selection of patients

All newly diagnosed and previously untreated asthmatic subjects in the moderate stage who were over than 15 years, were enrolled in this study. The study took place at outpatient pulmonary subspecialty clinic in Mashhad, Iran during 2014-2016. Criteria used for diagnosis of asthma included: 1- respiratory symptoms including cough, wheezing and/or dyspnea, positive history for airway hyperactivity and history of recurrent episodes; 2-spirometry that revealed obstructive patterns, Forced expiratory volume in one second (FEV1) in the range of 60-79% of predicted and showed significant improvement post bronchodilator. Subjects with severe forms of asthma were excluded. The subjects were also evaluated for a history of respiratory tract infections, bronchiectasis, sinusitis, vocal cord dysfunction (evaluated by the plateau in the flow volume curve or Maximal Expiratory Flow in 50% of vital capacity/ Maximal Inspiratory Flow in 50% of vital capacity% (MEF50/MIF50>2.2 (Sanz et al., 2013) and history of cigarette or water pipe smoking, and in case of positive findings, were excluded. All patients provided written informed consent. The study was continued until enough subjects (26 subjects in each group) were enrolled (Figure 1).

**Study design**

 The study was a prospective, randomized, double blinded, placebo-controlled, parallel-group clinical trial. All subjects gave their consent form and the study was approved by the ethic committee of Islamic Azad University- Mashhad branch (No. A/17/5/1393) and registered in the Iranian clinical trial registry (IRCT201209302695N4). 

Randomization and masking: the subjects were randomly divided into either propolis or placebo groups using the computer generated random Table. Assignment of subjects into each group was done by an independent assistant who was blinded to the groups.

Intervention: propolis as a dietary supplement was commercially available and prepared in the form of tablet (Propolis®, Soren Tech Toos Company, Mashhad, Iran). The propolis species in this preparation was Propolis Cera and it was the brown type and formulated as powder. The placebo was produced and packed identically in shape, color and size to the original drug (provided by the producer of Propolis®). The dosage was three tablets per day for one month. Each Propolis® tablet contained 75 mg. 

Blinding: the drug and placebo were coded by a non-dependent colleague and the drugs were prescribed by another pharmacist who was blinded to the code of the drug and placebo. This pharmacist kept the sealed code of the package until the end of the trial and the code was opened only in case of emergencies. Patients and investigators were unaware of assignments throughout the study. Inhaled salbutamol was allowed to be used as the choice reliever medication throughout the study. Outcome variables were evaluated by physicians and technicians who were unaware of the study groups.

Outcome variable measurement: primary end points were score improvement in the ACT questionnaire (Asthma Control Test, which is a valid questionnaire for evaluating the activity of asthma (Juniper et al., 1999)) and fraction of expiratory nitric oxide (FENO). FENO was measured by No Breath (Bedfont Medical instruments, London, England). Secondary end points were: frequencies of complete improvement of cough, dyspnea, and change in spirometry parameters: FEV1, FEV1/FVC, and fraction of inflammatory cells in sputum. Spirometry was done by a turbine spirometry device (Superspiro, Micomedical Company, London, UK) according to the American Thoracic Society/ European Respiratory Society guidelines (Miller et al., 2005). We also recorded incidence of side effects as the final step in the assessments.

Sputum induction: subjects were pre-medicated with two puffs (100 microgram per puff) of salbutamol in inhaler form to prevent bronchospasms by hypertonic saline. Sputum was induced by 5% saline inhalation that was carried out by a compressor-type nebulizer (CX3, Omron, Japan) according to European Respiratory Society guidelines (Djukanović et al., 2002). In each step, nebulization was performed for two minutes and after a 2-minute interval, if no sputum was expelled, the procedure was repeated three times. 

Sputum processing: a liquid base commercial kit (E-prep Plus sol, Tehran, Iran) was used for sputum preparation and decontamination of the saliva. Two slides were prepared and stained by Papanicolaou stain for each subject and 300 non-squamous cells were counted in each slide. The mean of the results of the two slides were recoded. For classification of inflammatory cells, subjects with eosinophilic percentage more than 3%, were classified as eosinophilic, neutrophilic percentage more than 76% as neutrophilic, eosinophilic and neutrophilic as mixed type, and none of them as paucigranulocytic (Sakai-Kashiwabara and Asano 2013; Simpson et al., 2006).

Time of outcome measurement: outcome variables were assessed at the first visit and over the thirty-day treatment period.

Follow up: telephone calls were used to follow the patients every two weeks. Any subject who complained of a noteworthy cough and unexpected side effect, were excluded from the study and he/she was asked to use other treatment managements. 

**Statistical analysis**

Sample size was calculated to provide 80% potency to detect 20% difference of primary outcome (ACT score) between the propolis and placebo groups by means of less than 5% error. Normal distribution was assessed by the Kolmogorov-Smirnov test. Comparison of outcome measurements between the propolis and placebo groups was made by the chi square, Fisher exact, Mann-Whitney U (for abnormally distributed subjects), and student’s t-tests (for normally distributed subjects). The results of treatment over the trial were analyzed by McNemar, and paired t-test. SPSS 19 software was used for statistical analysis. All hypothesis testing were two sided and significance was accepted at p<0.05.

 Role of funding source: The producer of Propolis® provided the drugs (propolis and placebos) with cost. Also, the company was not entered into the study results and had no role in the interpretation of our results.

## Results

**Basic demographical findings**

Fifty-two (20 female and 32 male) eligible patients were selected for the study. The patients who suffered from moderate stage asthma were recruited into this study and divided equally in two groups of propolis and placebo. Mean age was 44.6±18.5 years and most of them lived in the city and they were not exposed to air pollutions. Mean age and distribution of gender and occupation were not significantly different between the two groups. Four subjects (7%) reported air pollution in their work place, but the difference between two groups in this regard was not significant. Mean duration of asthma was 90±14.2 months (range: 1 month to 20 years) and the difference between the two groups was not significant (72±12 months in the propolis group and 107±13 months in placebo group, p=0.13). 

**Comparison of clinical findings**

Before the trial: the most frequent symptoms before the trial in both groups were cough, dyspnea and sputum production (Table 1). 

All subjects reported aggregation of symptoms after the exercise and night symptoms were mentioned in two-thirds of the subjects. A quarter of the subjects reported acute attack one month before. The mean attacks per week and emergency department visits per month were 2.6±2.6 and 0.92±0.2 respectively in the propolis group and 1.6±2.3 and 0.12±0.3 respectively in the placebo group, which were not significantly different. Gastero-esophageal reflux (GERD) and post-nasal drip (PND) were the most frequent accompanying symptoms in almost half of the subjects. The differences in the ACT score between the two groups before the trial were not significant (Table 2). After the trial: cough, dyspnea, wheeze, nocturnal symptoms and airway hyper-responsiveness were reduced significantly in the propolis group compared to the placebo group and the propolis group before the trial (Table 1). Propolis did not show any significant effect on sputum and symptoms after exercise. Acute attacks of asthma and emergency department visits were reduced 1.73 and 0.92 fold respectively by propolis. This was significantly lower than the placebo group, in which the attacks and emergency department visits were higher (0.06 and 0.18 folds respectively, p=0.001 and p=0.01 respectively). ACT score significantly improved in the propolis group (p<0.001) (Table 2), but in placebo group, the difference was not significant.

**Spirometry**

Before the trial: analysis of spirometry results before the trial showed a moderate obstructive pattern and no significant differences in this regard existed between the two groups (Table 2).

After the trial, almost all spirometry parameters increased significantly in the propolis group and in contrast all parameters decreased in the placebo group (Table 2). The propolis group also showed more significant improvement in almost all spirometry values compared to the placebo group.

**Table 1:** Comparison of demographic and clinical findings in the treatment of moderate persistent asthma by propolis and placebo tablet

	Total	Before trial		After trial	
		Propolis	Placebo	Propolis	Placebo
	N (%)	N (%)	N (%)	N (%)	N (%)
**Cough **	50 (96%)	25 (96%)	25 (96%)	7 (27%)*†	23 (88%)
**Dyspnea**	52 (100%)	26 (100%)	26 (100%)	10 (38%)*†	24 (94%)
**Wheeze**	52 (100%)	26 (100%)	26 (100%)	11 (42%)*†	24 (94%)
**Sputum**	42 (81%)	23 (88.5%)	19 (70%)	21 (80%)	23 (88%)
**Nocturnal Symptoms**	35 (67%)	20 (77%)	15 (58%)	5 (19%)*†	21 (82%)‡
**Acute attack**	13 (25%)	7 (27%)	4 (15%)	0 (0%)*†	4 (22%)
**AHR**	45 (86%)	22 (85%)	23 (88%)	13 (50%)*†	24 (94%)
**GERD**	24 (46%)	12 (46%)	12 (46%)	8 (31%)	14 (54%)
**PND**	29 (56%)	14 (56%)	15 (58%)	13 (50%)	17 (65%)

AHR=Airway hyper-responsiveness, GERD=Gastero-esophageal reflux, PND=Post- nasal drip

**p<0.05, **p<0.01, ***p<0.01=Significant difference between before and after the trial (McNemar test)

†=Significant difference between propolis and the placebo after the trial (chi square test)

‡=Significant difference between before and after the trial in the placebo group (McNemar test)

**Table 2:** Comparison of spirometry and physiological evaluation of subjects enrolled in the trial for the treatment of moderate persistent asthma by the propolis tablet

	Total	Before trial		After trial	
		Propolis	Placebo	Propolis	Placebo
	N (%)	N (%)	N (%)	N (%)	N (%)
**FVC (L)**	2.53±0.8	2.5±0.9	2.5±0.8	2.6±0.8	2.34±0.7‡
**FVC percent**	78.1±8.6	78.4±11.2	78±5	83.8±9.5*†	72.8±9.8‡
**FEV1 (L)**	1.84±0.64	1.85±0.56	1.82±0.56	2.03±0.7†	1.77±0.5‡
**FEV1 percent**	69.3±5.35	69.1±5.8	68.7±4.7	76±13.6*†	62.5±8.5‡
**FEV1/FVC**	73.5±7.1	74.7±9.3*	72.9±6.6	76.6±8.6*	74±13.3‡
**FEV1/VC**	81.2±1.6	81.5±14.4	77.5±10.3	85.5±19*	73.3±10.3‡
**FEF25-75 (L/S)**	1.45±0.5	1.4±0.5	1.4±0.4	1.85±0.9*†	1.3±0.5‡
**FEF25-75 percent**	39.6±8.1	40.7±9.66	37.6±5.11	49.1±21.3*†	34.4±8.4‡
**FEF25-75/FVC**	0.59±0.26	0.62±0.21	0.57±0.86	0.67±0.22*	0.55±0.13‡
**PEFR (L/S)**	2.17±0.86	1.9±0.8	2.4±0.9	2.3±0.9	1.9±0.6‡
**PEFR percent**	49.2±1.42	45±12.1†	54±15.1	53.1±14.2†	45.2±11.8‡
**FENO (PPM)**	61.9±6.4	64.2±52.5	63.3±46.4	42.9±43.3*†	83.2±80.3‡
**ACT**	13.4±5.4	12.8±5.5	13.9±5.09	19.4±4.39*†	12.8±6.2
**Acute attack**	2.14±2.56	2.64±2.67	1.65±2.4	0.73±0.17*	1.7±2.5
**ED V**	0.6±1.8	0.92±0.2	0.12±0.33	0*†	0.29±0.5

*=Significant difference after the trial compared to before treatment (paired t test)

†=Significant difference between propolis and the placebo after the trial (student t test or Mann-Whitney U test)

‡=Significant difference after the trial in the placebo group (paired t test)

FEV1=Forced expiratory volume in 1 second, FVC=Forced vital capacity, VC=Vital capacity, FEF25-75=Forced expiratory flow in 25- 75% of vital capacity, PEFR=Peak expiratory flow rate, FENO=Fraction of expiratory nitric oxide, ACT=Asthma control test score, EDV=Emergency department visit,

**Inflammatory parameters**

FENO: FENO in the propolis group it showed marked improvement after the trial (p<0.01). In contrast FENO increased significantly in the placebo group (p<0.05), (Table 2). The difference in FENO at the end of trial between the two groups was significant, (Table 2).

**Figure 2:**
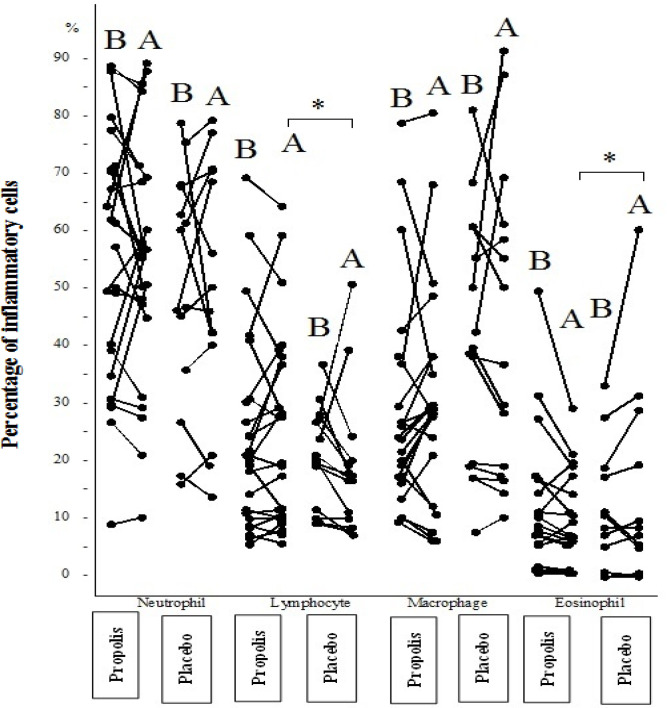
Inflammatory cells (%) in sputum before and after treatment with placebo and propolis in subjects suffering from moderate persistent asthma. B: before intervention; A: after intervention

Sputum inflammatory cells: neutrophil was the most frequently observed inflammatory cell in sputum of both propolis and placebo subjects but the difference between the two groups at the beginning of the study was not significant (Table 3). Eosinophil as the most important inflammatory cell was non-significantly higher in the propolis group (7.4±1.4%) than the placebo group (5.5±2.8%), before the trial. However, after the trial, the frequency of eosinophil was decreased significantly in the propolis group (4.3±3.1%, p=0.05) but increased in the placebo group (11.1±6.6%, p=0.05). In addition the frequency of eosinophil in the propolis group was significantly lower compared to the placebo group (p<0.05), (Figure 2). Frequency of lymphocyte was significantly increased in the propolis group after the trial (p=0.015) (Table 3). Neutrophil and macrophage did not change significantly after the trial (Figure 2 and Table 3). 

Although lymphocyte alteration during the trial was not significant in either group, it showed an increase in the propolis group after the trial (22.6±16.4%) causing a significant difference in lymphocyte frequency in the placebo group, which showed a significant decrease in lymphocyte (12.9±6.9%) (U=97, p=0.02) (Figure 2)

Eosinophilic pattern was the predominant pattern in both group, but its frequency and neutrophilic pattern decreased in both groups. In contrast, the paucigranulocytic pattern increased in the propolis group and mixed pattern increased in the placebo group (Table 3).

**Side effects**

Allergic skin rash was observed in two subjects in the propolis group, but this complication did not lead to the discontinuing of the treatment. However, an acute attack of asthma in one subject of the propolis group leading to discontinuing of the treatment who replaced with another one.

**Table 3:** Comparison of inflammatory cells in sputum of subjects enrolled in the trial for the treatment of moderate persistent asthma by the propolis tablet

	Total	Before trial		After trial	
		Propolis	Placebo	Propolis	Placebo
**Inflammatory cells in sputum**					
**Eosinophil (%)**	7.2±1.01	7.4±1.4	5.5±2.8	4.3±3.1*	11.1±6.6‡
**Lymphocyte (%)**	18.2±1.5	18.5±17.3	16.2±8.7	22±1.6†	12.9±6.9
**Neutrophil (%)**	47.2±2.4	47.7±25.5	45.4±21.4	47.2±23	42.4±26.3
**Macrophage (%)**	27.2±2.3	26.5±25.6	32.3±21.9	26.5±21.1	33.5±27.4
**Cytological pattern of inflammation**					
**Eosinophilic N(%)**	23 (44%)	11 (41%)	12 (46%)	10 (38%)	8 (31%)
**Mixed N(%)**	4 (8%)	2 (8%)	2 (8%)	1 (4%)	4 (15%)
**Neutrophilic N (%)**	10 (19%)	5 (19%)	5 (19%)	3 (12%)	4 (15%)
**Paucigranulocytic N(%)**	15 (29%)	8 (31%)	7(27%)	10 (38%)	8 (31%)
**No sputum N(%)**	0	0	0	2 (8%)	2 (8%)

*=Significant difference after the trial in the propolis group

†p<0.05; Significant difference between propolis and the placebo after the trial.

‡p<0.05; Significant difference after the trial in the placebo group (paired t test).

## Discussion

This study was a prospective double blind placebo controlled clinical trial on the effect of propolis (a by-product of the honey bee) on moderate persistent asthma. 

Results of the study showed that 75 mg of propolis three times daily for one month was able to suppress major clinical findings of asthma including cough, dyspnea, airway hyper-responsiveness, and anight symptoms and improve asthma control as shown by the ACT score. Physical exam revealed significant improvement of wheezing and frequency of acute asthmatic attacks that required an emergency room visit or drug usage. Propolis inhibited inflammation and reduced FENO and it was able to improve the respiratory physiology of asthma by increasing spirometry parameters including FEV1 and mid-expiratory flow parameters. 

Although strong evidence exists in folk medicine about the potential effect of propolis, documented clinical experience on the effect of honey and propolis was not reported. In addition, the proposed mechanism of action of propolis on asthma has yet to be described. The effect of propolis on the immune system is the most well-known mechanism (Sforcin 2007). Experimental studies showed that propolis suppressed Th1 and Th2 activity by a new mechanism: Erk2 MAP-kinase signal pathway (Burdock 1998). In that study, propolis was able to suppress many mediators including IL-1, IL-12, IL-2, Transforming growth factor (TGF), IL10, IL4, but it induced TGF-b. Other studies indicated that caffeic acid phenethyl ester showed an inhibitory effect on monocyte-derived dendritic cells as a leading cell in asthma production and triggering (Wang et al., 2009). Propolis also showed a wonderful effect on asthma by suppressing eotaxin and IL-13; therefore it has strong potential for, inhibiting eosinophil chemotaxis (Liao et al., 2010). Therefore, propolis is able to extensively regulate asthma.

Propolis also showed potent antimicrobial and anti-fungal activity (Burdock 1998). This mechanism may open a new method in treating asthma for physicians who suspect infection has a potential role in asthma and who have a tendency towards radical treatments of asthma by eradication of infection.

Clinical studies on the effect of propolis on asthma are very limited. Khayyal et al. (1993) performed a clinical experiment similar to the present study (Khayyal et al., 1993). This study included 46 subjects and the most important difference with our present study was the inclusion of another subject category (mild persistent asthma) and more elongated course of therapy (two-month period). Comparable to this study, clinical findings, frequency of acute attacks and spirometry parameters improved significantly. The present study evaluated FENO as a crude evaluation of inflammation in asthma that was not determined by Khayyal et al. (1993). But in Khayyal et al. study, the evaluation of mediators was included. 

In current study propolis was able to suppress TNF-alpha, ICAM-1, IL-6 and IL-8, PG-E2, F2 alpha, D4 and caused a 3-fold increase in the 'protective' cytokine IL-10. These findings indicated the potential effect of propolis on lung inflammation in asthma. The present study repeated these remarkable results and it is recommended to continue this type of research with larger clinical studies and new methodologies to determine the preference of prescribing propolis, either alone or in combination with inhaled corticosteroids. 

Although this study was not a pioneer study on propolis, it is a rare study about the efficacy of propolis on asthma. The subjects were randomly enrolled into two similar groups and treated similarly. The patients, clinicians, and personnels were blinded to the treatment. The drug (Propolis®) and placebo were produced by the company in a similar fashion. A subject discontinued the propolis, but all other 51 subjects finished the 30-day course of the study. Therefore, this attrition has not materially impacted the results of this study. Analysis of the study was performed by standard parameters usually used in clinical trials. This study showed the efficacy of propolis for the treatment of asthma as a controller drug. The placebo effect was not seen, as asthma was worsened in the placebo group. Inflammatory cells were not markedly changed, but FENO, as a marker of inflammation in asthma, improved significantly. Therefore, propolis may have an effect via the mediators' release. In this regard, further investigations on the exact mechanism of action of propolis and its ingredients on asthma would be beneficial. More molecular studies would be able to reveal a new spectrum on the effect of propolis ingredients on T helper 2 response (such as IL-4, Il-5 and IL-13) and T helper 17 (such as IL-17) and Toll like-2 receptor. The effect of propolis on genetic signaling of asthma including the nuclear factor kappa-b was introduced before (Márquez et al., 2004). In this regard, it was proposed that propolis is able to control asthma for a long time. Therefore, further research would be able to determine the efficacy of propolis in a long term period compared inhaled corticosteroid plus long acting beta 2 agonist. 

Treatment with propolis showed improvement of clinical and physiological parameters of asthma which indicate its potential effect on the treatment of asthma. 
